# Therapeutic Advances in *Mycobacterium avium* Complex Pulmonary Disease: Current Treatments and Emerging Host-Directed Approaches

**DOI:** 10.3390/ijms27219872

**Published:** 2026-11-04

**Authors:** Nooneh Melody Khachatourian, Rubina Takhmazian, Vishwanath Venketaraman

**Affiliations:** College of Osteopathic Medicine of the Pacific, Western University of Health Sciences, 309 E. Second St., Pomona, CA 91766-1854, USA

**Keywords:** *Mycobacterium avium* complex, non-tuberculous mycobacteria, MAC pulmonary disease, host-directed therapy, glutathione, antimicrobial resistance, intracellular persistence, multidrug therapy, emerging therapeutics

## Abstract

*Mycobacterium avium* complex (MAC) is the leading cause of non-tuberculous mycobacterial (NTM) pulmonary disease, with increasing incidence among aging and immunocompromised populations. Despite guideline-recommended multidrug therapy consisting of a macrolide, ethambutol, and a rifamycin, treatment remains prolonged and complicated by incomplete culture conversion, adverse effects, recurrence, and antimicrobial resistance. Intracellular persistence, biofilm formation, and intrinsic resistance mechanisms further challenge disease management. This review summarizes MAC pathogenesis, host immune responses, current treatments, and emerging therapeutic strategies. Promising approaches include novel antimicrobial agents, optimized multidrug regimens, drug repurposing, inhaled delivery systems, and host-directed therapies. Particular emphasis is placed on glutathione (GSH), an intracellular antioxidant with immunomodulatory properties, as a potential adjunctive therapy. Experimental studies suggest that GSH and its precursor N-acetylcysteine may influence oxidative stress, inflammatory responses, and antimycobacterial activity. However, MAC-specific evidence remains primarily in vitro, and clinical efficacy in MAC pulmonary disease has not been established. Findings from related mycobacterial infections provide mechanistic insights but cannot be directly extrapolated to MAC pulmonary disease. Further preclinical and clinical studies are needed to determine whether GSH-based interventions can safely complement conventional antimicrobial therapy and improve treatment outcomes.

## 1. Introduction

Non-tuberculous mycobacteria (NTM) comprise a diverse group of environmental mycobacteria distinct from *Mycobacterium tuberculosis* (*M. tuberculosis*) complex and *Mycobacterium leprae* [1]. These organisms are commonly found in soil, natural water, municipal water systems, and other environmental reservoirs, making human exposure frequent and difficult to prevent [1,2]. More than 190–200 NTM species have been described, although only a subset are consistently associated with clinically significant human disease [1,3]. NTM infections most commonly involve the lungs, but disease may also affect lymph nodes, skin and soft tissue, bone, joints, and, in immunocompromised hosts, may become disseminated [3].

The incidence and prevalence of NTM pulmonary disease have increased globally over recent decades, particularly in North America, Europe, and Asia. This rise has been attributed to multiple factors, including aging populations, increased prevalence of chronic lung disease, expanding immunosuppressed populations, improved microbiological diagnostics, and greater clinical awareness [1,3]. Unlike tuberculosis, NTM disease is not uniformly reportable, making true epidemiologic burden difficult to define and likely underestimated in many regions [1,2]. Diagnosis is also complicated by the fact that isolation of NTM from respiratory samples does not always indicate active disease, requiring integration of clinical symptoms, radiographic abnormalities, and microbiological criteria [3].

Among pathogenic NTM, *Mycobacterium avium* complex (MAC) is the leading cause of NTM lung disease worldwide [2,4]. MAC includes clinically important species such as *M. avium*, *M. intracellulare*, and *M. chimaera*, with species distribution varying by geographic region [2]. MAC pulmonary disease typically presents as either nodular-bronchiectatic disease, often seen in older individuals and patients with bronchiectasis, or fibrocavitary disease, which may resemble tuberculosis or malignancy and is associated with more severe outcomes [2,3]. Susceptibility to MAC disease is strongly influenced by host factors, including bronchiectasis, chronic obstructive pulmonary disease, cystic fibrosis, prior tuberculosis, low body mass index, immunosuppressive therapy, HIV/AIDS, and defects in the IL-12–IFN-γ axis [3].

The clinical significance of MAC lies not only in its frequency but also in its chronicity, treatment complexity, and impact on quality of life [2]. Patients may experience persistent cough, sputum production, fatigue, weight loss, dyspnea, hemoptysis, and recurrent pulmonary exacerbations [3]. Even when disease progresses slowly, MAC infection can contribute to structural lung damage and prolonged morbidity [2,3]. In immunocompromised individuals, particularly those with advanced HIV infection, MAC may cause disseminated disease, further increasing clinical burden [3].

MAC pulmonary disease is generally treated with prolonged multidrug antibiotic therapy. For macrolide-susceptible MAC pulmonary disease, the standard regimen consists of a macrolide, ethambutol, and a rifamycin, continued for at least 12 months after sputum culture conversion. In contrast, macrolide-resistant MAC pulmonary disease requires an individualized treatment approach, often involving additional or alternative antimicrobial agents [3,5]. Despite guideline-based therapy, outcomes remain suboptimal: treatment is lengthy, adverse effects are common, and culture conversion rates vary widely [1,3]. Standard therapy for MAC pulmonary disease frequently extends over 18 months, with reported sputum culture conversion rates of approximately 45–70% across heterogeneous studies. Even after an initial successful response, recurrent disease remains a challenge, although reported recurrence rates vary substantially with patient characteristics, follow-up duration, and whether relapse and reinfection are distinguished [1,5,6].

Drug resistance is a central barrier to successful MAC treatment. NTM possess intrinsic resistance mechanisms, including a lipid-rich, relatively impermeable cell wall, slow growth, intracellular persistence, efflux activity, and biofilm formation [1,2]. In MAC lung disease, macrophage residence and airway biofilms can limit antibiotic penetration and contribute to chronic infection [2]. Among available drugs, macrolide susceptibility is one of the strongest predictors of treatment success, while macrolide resistance is associated with poor outcomes and increased mortality [3]. However, drug susceptibility testing remains imperfect; phenotypic testing correlates best with clinical outcomes for macrolides and amikacin, whereas correlations are weaker for other drugs such as rifampin and ethambutol [1,7].

These limitations highlight the need for improved therapeutic strategies for MAC and other NTM infections. Current challenges include prolonged treatment duration, toxicity, poor adherence, limited drug pipelines, recurrent disease, biofilm-associated tolerance, and emergence of antimicrobial resistance [1,4,5]. Accordingly, drug discovery efforts are increasingly focused on optimized combinations, repurposed antimicrobials, inhaled formulations such as amikacin liposome inhalation suspension, novel small molecules, host-directed therapies, and adjunctive approaches that may improve bacterial clearance while reducing toxicity [4,5]. The biological complexity of MAC infection, characterized by intracellular persistence, chronic inflammation, antimicrobial tolerance, and prolonged treatment requirements, has shifted therapeutic research beyond conventional antibiotics toward integrated antimicrobial and host-directed strategies. This review explores current treatment modalities for MAC while highlighting emerging therapeutic approaches, with particular emphasis on GSH as a potential adjunctive host-directed therapy.

Although recent reviews have examined the challenges of MAC pulmonary disease management and advances in antimicrobial development, important questions remain regarding the integration of conventional antimicrobial therapy with emerging host-directed approaches. This review provides an integrated assessment of current guideline-based treatments, novel antimicrobial agents, drug repurposing, and host-directed therapeutic strategies, with particular emphasis on glutathione (GSH) and its precursor N-acetylcysteine (NAC). Rather than focusing solely on potential therapeutic benefits, we critically examine the available evidence for GSH- and NAC-based interventions, distinguishing MAC-specific experimental findings from evidence derived from tuberculosis and other mycobacterial models. We also highlight the limitations of existing studies and identify research priorities necessary to determine the clinical applicability of these approaches in MAC pulmonary disease.

### Literature Search Strategy

This narrative review was informed by a literature search of PubMed covering publications from January 2018 through 18 October 2026. Search terms included “*Mycobacterium avium* complex,” “MAC pulmonary disease,” “nontuberculous mycobacteria,” “treatment,” “antimicrobial resistance,” “host-directed therapy,” “glutathione,” and “N-acetylcysteine,” used individually and in relevant combinations. Priority was given to clinical practice guidelines, randomized clinical trials, systematic reviews, meta-analyses, and experimental studies directly relevant to MAC pulmonary disease. Earlier landmark publications were also considered when necessary to provide historical context or establish current treatment recommendations. Because MAC-specific evidence for certain emerging therapies remains limited, selected studies involving disseminated MAC infection, other NTM species, and *Mycobacterium tuberculosis* were included to provide mechanistic or therapeutic context. Findings from these populations and organisms were distinguished from direct MAC-PD evidence and were not assumed to establish clinical efficacy in pulmonary MAC disease. Literature selection was narrative rather than systematic; therefore, no formal risk-of-bias assessment or quantitative evidence synthesis was undertaken.

The primary focus of this review is treatment of pulmonary disease caused by MAC, particularly in adults with chronic structural lung disease. Evidence from disseminated MAC infection is considered separately because differences in host immune status, infection burden, and disease manifestations may limit its applicability to MAC-PD. Similarly, findings from tuberculosis and other NTM infections are discussed only where they inform relevant antimicrobial mechanisms or host-directed therapeutic hypotheses. Throughout this review, direct clinical evidence in MAC-PD is distinguished from MAC-specific laboratory findings and extrapolations from other mycobacterial diseases.

## 2. Pathogenesis and Host Immune Response

MAC pulmonary disease develops following inhalation of aerosolized organisms from environmental reservoirs, including water distribution systems and soil, allowing bacteria to reach the distal airways and alveolar spaces where they are rapidly engulfed by resident macrophages [1,8]. In most immunocompetent individuals, innate and adaptive immune responses successfully limit infection. However, in susceptible hosts—particularly those with impaired mucociliary clearance, chronic structural lung disease, or immune dysfunction—MAC establishes persistent intracellular infection that promotes chronic inflammation and progressive pulmonary injury [3,9].

### 2.1. Virulence and Intracellular Survival

The ability of *M. avium* to survive within macrophages is fundamental to its pathogenicity. Following phagocytosis, MAC actively interferes with normal phagosomal maturation, preventing efficient fusion between phagosomes and lysosomes and thereby avoiding exposure to degradative enzymes and acidic intracellular compartments [8,10]. Rather than being eliminated, the bacterium creates a protected intracellular niche where replication and long-term persistence can occur. Several bacterial proteins have been implicated in these processes by modifying vesicular trafficking and host cell signaling, ultimately favoring bacterial survival over macrophage antimicrobial activity [10].

In addition to disrupting intracellular trafficking, MAC possesses robust defense mechanisms against oxidative and nitrosative stress generated by activated phagocytes. Macrophages normally produce reactive oxygen species (ROS) and reactive nitrogen species (RNS) as part of their antimicrobial arsenal, yet *M. avium* expresses antioxidant enzymes—including superoxide dismutases, catalase, and alkyl hydroperoxide reductase—that detoxify these reactive intermediates before they can damage essential bacterial components. Redox-regulated repair systems further protect proteins and other macromolecules from oxidative injury, enabling MAC to withstand prolonged residence within activated macrophages [8]. These adaptive responses illustrate why intracellular persistence remains a major obstacle to successful antimicrobial therapy.

The lipid-rich mycobacterial cell envelope provides an additional layer of protection. The highly hydrophobic cell wall restricts antibiotic penetration while simultaneously contributing to resistance against host-derived antimicrobial molecules [1,11]. Beyond the individual bacterial cell, MAC can organize into biofilms that enhance survival in both environmental reservoirs and the respiratory tract [11]. Biofilm formation promotes bacterial aggregation, limits antimicrobial penetration, and increases tolerance to host immune responses, thereby facilitating long-term colonization and recurrent infection [11]. These characteristics likely contribute to the high rates of relapse and reinfection observed even after prolonged antimicrobial treatment [5].

### 2.2. Immune Dysregulation and Chronic Inflammation

Protective immunity against MAC relies predominantly on a coordinated Th1 immune response. Recognition of mycobacterial pathogen-associated molecular patterns by pattern-recognition receptors on macrophages and dendritic cells initiates signaling cascades that stimulate production of IL-12, TNF-α, and other pro-inflammatory cytokines, ultimately promoting IFN-γ production by T lymphocytes and natural killer cells [9,10]. IFN-γ is particularly important because it enhances macrophage antimicrobial activity, stimulates phagolysosomal maturation, and promotes intracellular killing of mycobacteria [10]. Defects in the IL-12/IFN-γ signaling axis therefore markedly increase susceptibility to disseminated MAC disease, underscoring the importance of cell-mediated immunity in controlling mycobacterial infection [9]. Although these findings provide insight into protective immune mechanisms, the immunological and clinical characteristics of disseminated MAC disease differ from those of MAC pulmonary disease.

Despite activation of these immune pathways, persistent intracellular infection frequently results in dysregulated inflammation rather than bacterial clearance. Continuous recruitment of macrophages, neutrophils, and lymphocytes sustains granulomatous inflammation within the lung, where granulomas function to contain infection but may also become sites of ongoing tissue injury when sterilization is not achieved [9]. Over time, persistent inflammatory responses contribute to airway remodeling, bronchiectasis, cavitary lesions, and progressive decline in pulmonary function that characterize chronic MAC pulmonary disease [3,9].

The outcome of MAC infection is influenced by macrophage cell-death pathways and intracellular stress responses. Apoptosis is a regulated form of cell death that can contribute to host defense, whereas necrotic cell death may facilitate bacterial release and local inflammation [12]. In contrast, autophagy is primarily a catabolic, stress-adaptive process that promotes the degradation and recycling of intracellular components and can contribute to the clearance of intracellular pathogens. Although autophagy-dependent cell death has been recognized as a distinct phenomenon, autophagy itself should not be classified as a programmed cell-death pathway [13]. Evidence from mycobacterial infection models, including studies involving Mycobacterium tuberculosis, suggests that these processes may influence intracellular bacterial persistence. However, their specific roles in MAC infection require further investigation before autophagy modulation can be established as a therapeutic strategy [10,12].

Host susceptibility further shapes disease progression. Individuals with bronchiectasis, chronic obstructive pulmonary disease, cystic fibrosis, prior pulmonary tuberculosis, HIV infection, or other forms of immunosuppression exhibit impaired airway clearance and diminished immune function, creating conditions that favor persistent MAC infection [3,9]. These host factors interact with bacterial virulence mechanisms to establish a cycle of intracellular persistence, chronic inflammation, and progressive lung damage that is difficult to interrupt using antibiotics alone.

**Figure 1:**
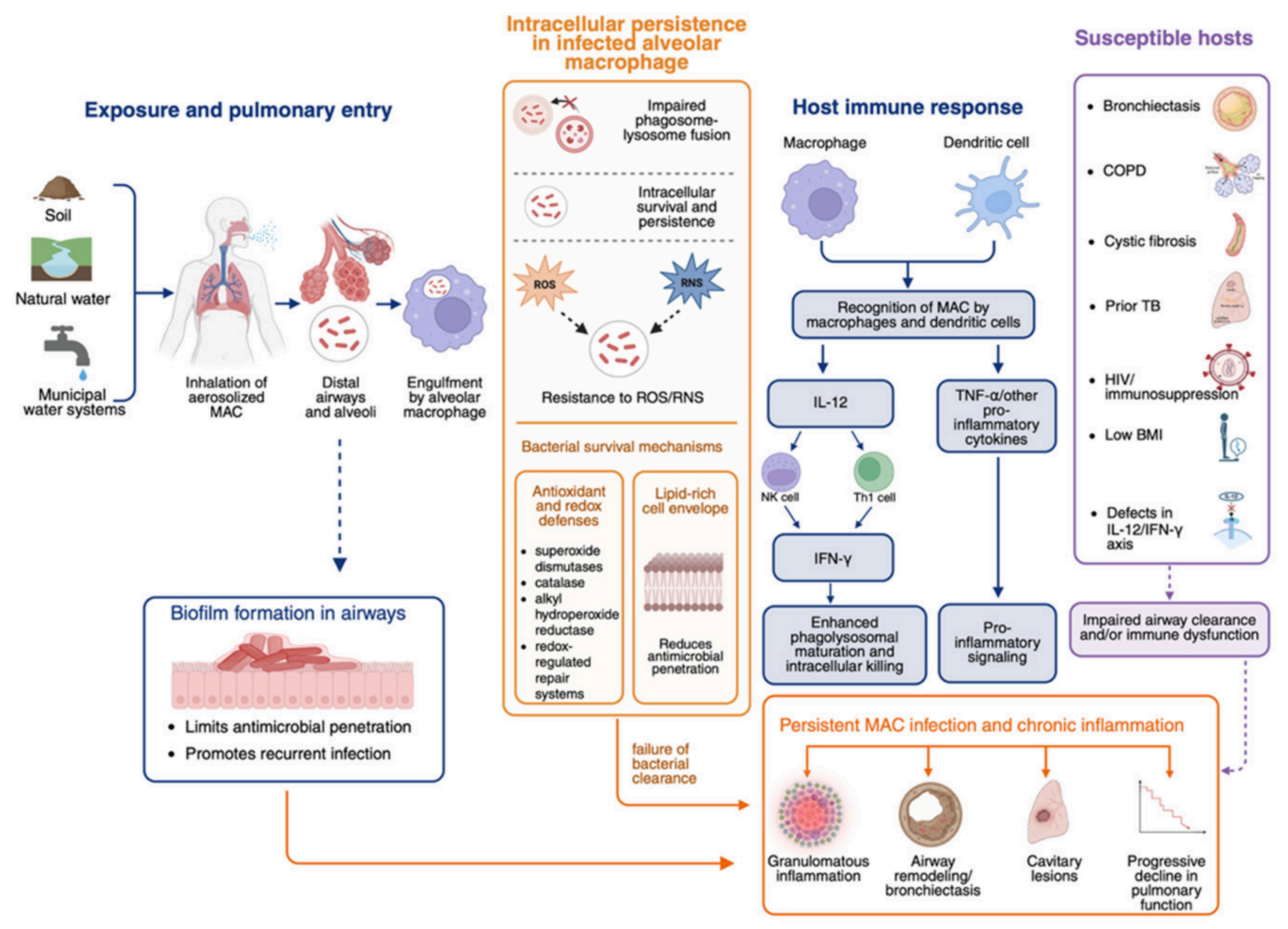
Pathogenesis and host immune response in *Mycobacterium avium* complex pulmonary disease. MAC organisms found in soil, natural water or municipal water systems may enter the respiratory tract through inhalation of aerosolized bacteria and reach the distal airways and alveoli. After uptake by alveolar macrophages, MAC can inhibit phagosome-lysosome fusion and resist reactive oxygen species (ROS) and reactive nitrogen species (RNS) through antioxidant enzymes and redox repair systems. Its lipid-rich cell envelope and ability to form airway biofilms further support survival and persistence. Recognition of MAC by macrophages and dendritic cells induces IL-12, TNF-α, and other pro-inflammatory cytokines. IL-12 stimulates IFN-γ production by natural killer and Th1 cells, which enhances phagolysosomal maturation and intracellular killing. In susceptible hosts, including individuals with bronchiectasis, chronic obstructive pulmonary disease, cystic fibrosis, prior tuberculosis, HIV or other forms of immunosuppression, low body mass index, or defects in the IL-12/IFN-γ axis, impaired airway clearance or immune responses may allow infection to persist. Chronic infection and inflammation can lead to granulomatous inflammation, airway remodeling and bronchiectasis, cavitary diseases, and progressive loss of pulmonary function. Solid arrows show the main pathway, and dashed arrows show contributing relationships. Created in BioRender. Takhmazian, R. (2026) https://BioRender.com/4xah638.

Collectively, current evidence suggests that MAC pathogenesis reflects a dynamic interaction between bacterial survival strategies and host immune dysfunction. Intracellular persistence, resistance to oxidative killing, biofilm formation, and immune modulation allow *M. avium* to evade eradication, while chronic inflammatory responses contribute substantially to pulmonary tissue destruction [10,11]. Understanding these mechanisms provides the biological rationale for developing therapies that extend beyond direct antimicrobial activity to include host-directed interventions aimed at restoring effective immune function and improving treatment outcomes. These major pathogenic pathways are summarized in Figure 1.

## 3. Current Treatment Modalities

Current treatment of *M. avium* complex pulmonary disease (MAC-PD) remains challenging because therapy must eradicate an intracellular pathogen that exhibits intrinsic antimicrobial resistance, slow growth, and prolonged persistence within structurally abnormal lungs [1,3]. Treatment typically extends for 18–24 months from initiation and continues for at least 12 months after sputum culture conversion [3,5,6]. Although current guideline-based regimens have improved clinical outcomes compared with historical monotherapy, treatment success remains inconsistent and is frequently limited by adverse drug reactions, poor adherence, recurrence, and the emergence of antimicrobial resistance [1,3,5].

### 3.1. Standard Multidrug Therapies

International ATS/ERS/ESCMID/IDSA guidelines recommend a macrolide-based multidrug regimen as the cornerstone of therapy for patients with macrolide-susceptible MAC-PD [3,6,14]. The preferred regimen consists of either azithromycin or clarithromycin in combination with ethambutol and a rifamycin, typically rifampin or rifabutin [3,6]. Azithromycin is generally favored because of its lower potential for drug–drug interactions and improved tolerability compared with clarithromycin [6]. Patients with non-cavitary nodular bronchiectatic disease are commonly treated using intermittent (three-times-weekly) therapy, whereas those with fibrocavitary disease, extensive nodular bronchiectasis, or severe pulmonary involvement generally require daily treatment to maximize microbiological response [3,6].

For patients with cavitary MAC pulmonary disease, advanced or severe bronchiectatic disease, or macrolide-resistant MAC infection, current ATS/ERS/ESCMID/IDSA guidelines suggest adding parenteral amikacin or streptomycin to the initial antimicrobial regimen, generally for approximately 2–3 months, with careful monitoring for ototoxicity and nephrotoxicity [3,6,14]. This recommendation should be distinguished from the role of amikacin liposome inhalation suspension (ALIS), which is not recommended as part of initial therapy for newly diagnosed MAC-PD. Rather, ALIS is recommended as an addition to guideline-based multidrug therapy in patients who remain sputum culture-positive after at least six months of treatment [3,6]. In the randomized CONVERT trial involving treatment-refractory MAC-PD, sputum culture conversion by month six occurred in 29.0% of patients receiving ALIS plus guideline-based therapy compared with 8.9% receiving guideline-based therapy alone (odds ratio, 4.22; 95% confidence interval, 2.08–8.57) [15]. Respiratory adverse events were more frequent in the ALIS group (87.4% versus 50.0%), highlighting tolerability concerns [15]. Although inhaled liposomal delivery is intended to increase local pulmonary exposure and reduce systemic exposure, the CONVERT trial did not directly compare ALIS with intravenous amikacin and therefore cannot establish superior safety relative to parenteral administration.

Ethambutol remains a critical component of multidrug therapy because it reduces the likelihood of acquired macrolide resistance during prolonged treatment [6]. Preservation of macrolide activity is particularly important because macrolides represent the only drug class consistently associated with improved microbiological outcomes in MAC infection [3]. Consequently, treatment modifications that eliminate ethambutol without an effective substitute may substantially increase the risk of resistance development [6]. The decision to initiate antimicrobial therapy should be individualized rather than based solely on microbiological isolation or fulfillment of diagnostic criteria. In selected patients with mild, nonprogressive disease, observation with regular clinical, radiological, and microbiological monitoring may be appropriate after consideration of symptoms, disease severity, progression risk, comorbidities, and patient preferences [3,6]. Conversely, treatment is generally favored in patients with cavitary disease, positive acid-fast bacilli sputum smears, or progressive clinical manifestations. Surgical resection may also be considered as an adjunct to antimicrobial therapy in carefully selected patients, particularly those with localized disease, refractory infection, or complications such as significant hemoptysis, following multidisciplinary assessment of operative risk and expected benefit [3,6].

### 3.2. Treatment Outcomes and Limitations

Despite prolonged multidrug therapy, treatment outcomes in MAC pulmonary disease remain suboptimal. Importantly, treatment success, sputum culture conversion, and post-treatment recurrence represent distinct outcomes and should not be interpreted interchangeably. Sputum culture conversion generally refers to the achievement of at least three consecutive negative sputum cultures collected at least four weeks apart during treatment. Treatment success is a broader outcome that depends on the definition applied in the individual study and may incorporate sustained microbiological response and completion of the prescribed treatment course. Recurrence refers to the reappearance of MAC disease after an initial successful response and may represent either relapse involving the original infecting strain or reinfection with a different strain acquired from the environment. Reported recurrence rates vary according to the study population, microbiological definitions, and duration of follow-up. Molecular epidemiologic studies suggest that reinfection accounts for a substantial proportion of recurrent episodes, particularly in patients with persistent structural lung disease [1,5,6].

Treatment duration itself contributes substantially to poor clinical outcomes. Patients often require therapy for well over a year after culture conversion, resulting in total treatment courses approaching 18–24 months [3,5]. Such prolonged regimens place considerable burdens on patients and contribute to declining adherence over time. Premature discontinuation, treatment interruption, or non-guideline prescribing have all been associated with poorer microbiological outcomes and increased risk of macrolide resistance [6]. Furthermore, underlying structural lung disease frequently persists even after microbiological clearance, predisposing patients to recurrent infection despite apparently successful treatment [3].

Recent studies have investigated whether selected patients with MAC pulmonary disease may be treated effectively with two-drug regimens instead of conventional three-drug therapy. A systematic review and meta-analysis comparing these approaches found no statistically significant difference in overall bacteriological response between two-drug and three-drug regimens for MAC pulmonary disease (odds ratio [OR], 0.82; 95% confidence interval [CI], 0.53–1.25; *p* = 0.35), or in acquired macrolide resistance (risk difference [RD], 0.01; 95% CI, −0.02 to 0.05; *p* = 0.39) [16]. In subgroup analyses, macrolide–ethambutol regimens also showed no statistically significant difference in bacteriological response (OR, 1.54; 95% CI, 0.78–2.93; *p* = 0.23) or acquired macrolide resistance (RD, 0.01; 95% CI, −0.02 to 0.04; *p* = 0.50) compared with three-drug regimens [16]. However, the absence of statistically significant differences does not establish therapeutic equivalence or noninferiority, particularly given the limited available evidence and uncertainty reflected in the confidence intervals. Furthermore, findings from disseminated MAC infection should not be directly extrapolated to pulmonary disease; the same meta-analysis identified an increased risk of acquired macrolide resistance with two-drug therapy in disseminated MAC infection (OR, 2.99; 95% CI, 1.10–8.13; *p* = 0.03) [16]. Current ATS/ERS/ESCMID/IDSA guidelines continue to favor a three-drug macrolide-containing regimen, including ethambutol and a rifamycin, for macrolide-susceptible MAC pulmonary disease [3,6,14]. Although simplified regimens may warrant further investigation in carefully selected patients, existing findings do not justify broadly replacing guideline-recommended three-drug therapy.

### 3.3. Drug Toxicity and Resistance

Treatment-associated toxicity represents one of the principal barriers to successful MAC management. Macrolides are generally well tolerated but may produce gastrointestinal intolerance, hepatotoxicity, hearing impairment, and QT interval prolongation, particularly when combined with other cardiotoxic medications [3]. Ethambutol is associated with dose-dependent optic neuritis, necessitating routine ophthalmologic monitoring throughout treatment [6]. Rifampin and rifabutin frequently cause hepatotoxicity and potent induction of cytochrome P450 enzymes, creating clinically important drug–drug interactions that complicate treatment in patients receiving immunosuppressive agents, antiretroviral therapy, or other chronic medications [3]. Aminoglycosides, although effective against severe disease, are limited by nephrotoxicity and irreversible ototoxicity, restricting their duration of use and emphasizing the value of inhaled formulations such as ALIS [5,6].

In addition to toxicity, intrinsic and acquired antimicrobial resistance substantially limit therapeutic success. MAC possesses a highly impermeable, lipid-rich cell envelope, active efflux systems, biofilm-forming capacity, and metabolic adaptations that collectively reduce antimicrobial susceptibility [1,11]. These intrinsic characteristics reduce antimicrobial penetration and contribute to prolonged bacterial persistence despite multidrug therapy [11]. Acquired resistance most commonly develops against macrolides, frequently following inappropriate monotherapy, interruption of companion drugs, or incomplete multidrug regimens [6]. Once macrolide resistance emerges, treatment success declines dramatically, often to below 20–30% in published series, because few alternative agents demonstrate comparable clinical efficacy [3,6].

The interpretation of antimicrobial susceptibility testing also presents challenges. Current guidelines recommend routine susceptibility testing primarily for clarithromycin and amikacin because these agents demonstrate the strongest relationship between in vitro susceptibility and clinical response. For many other antimicrobials, including rifampin, ethambutol, fluoroquinolones, and linezolid, the relationship between minimum inhibitory concentrations and therapeutic outcomes remains inconsistent. Furthermore, substantial interlaboratory variability in broth microdilution testing has been reported, reflecting both methodological differences and biological diversity among MAC lineages [10]. These limitations complicate individualized therapy and reinforce the need for improved microbiological resistance markers and more predictive susceptibility testing platforms.

Collectively, current multidrug regimens have significantly improved the management of MAC-PD; however, prolonged treatment duration, incomplete culture conversion, recurrent disease, cumulative toxicity, and persistent antimicrobial resistance continue to compromise patient outcomes [1,3,5,6,7,16]. These shortcomings highlight the need for innovative therapeutic strategies that not only improve antimicrobial activity but also shorten treatment duration, reduce toxicity, and overcome mechanisms of bacterial persistence. Such unmet needs have driven increasing interest in novel antimicrobial agents, host-directed therapies, optimized drug delivery systems, and drug repurposing approaches that constitute the next generation of MAC therapeutics.

## 4. Emerging Therapeutic Approaches

To address the limitations of existing MAC therapies, current research is exploring complementary strategies, including antibiotic optimization, drug repurposing, novel antimicrobial targets, host-directed therapies (HDTs), and advanced drug delivery systems [4,5,17].

### 4.1. Novel Antimicrobial Agents

The development of novel and repurposed antimicrobial agents has generated interest in expanding treatment options for MAC pulmonary disease. Several compounds target bacterial pathways involved in energy metabolism, DNA replication, or protein synthesis, potentially offering mechanisms distinct from those of conventional MAC antimicrobials. However, their therapeutic potential must be evaluated according to the strength of MAC-specific evidence, including experimental activity, clinical outcomes, safety, and limitations of available studies [4,17].

Bedaquiline, a diarylquinoline that inhibits mycobacterial ATP synthase, has demonstrated antimycobacterial activity in experimental models and has been investigated as a potential treatment option for difficult-to-treat MAC infection [4,6]. Its mechanism of action disrupts bacterial energy metabolism, providing a potential alternative to conventional antimicrobial targets. Nevertheless, clinical evidence supporting its efficacy in MAC pulmonary disease remains limited. Safety concerns, including QT interval prolongation and potential drug interactions, also require consideration. Bedaquiline should therefore be regarded as an investigational or selectively considered salvage option rather than an established component of standard MAC-PD therapy.

Clofazimine, a riminophenazine antimicrobial, has been investigated as an alternative companion drug in MAC pulmonary disease [4,6]. In a single-center, nonblinded randomized trial involving 40 patients, Zweijpfenning et al. compared a clofazimine-ethambutol-macrolide regimen with a rifampicin-ethambutol-macrolide regimen. At six months, sputum culture conversion occurred in 62% (13/21) of patients receiving clofazimine and 58% (11/19) receiving rifampicin in the intention-to-treat analysis, while on-treatment conversion was 79% in both groups [18]. Treatment discontinuation occurred in 33% and 26% of patients, respectively. Diarrhea was more frequent in the clofazimine group (76% versus 37%; *p* = 0.012), whereas arthralgia was more frequent in the rifampicin group (37% versus 5%; *p* = 0.011) [18]. Although these findings suggest that clofazimine may be a reasonable alternative to rifampicin in selected patients, the small sample size and nonblinded, single-center design limit definitive conclusions regarding comparative effectiveness.

SPR720 is an investigational oral aminobenzimidazole that is converted to SPR719, an active compound that inhibits the ATPase activity of mycobacterial DNA gyrase B (GyrB), thereby interfering with DNA replication [19]. Although preclinical studies supported its potential activity against MAC, results from a randomized phase 2 study were less encouraging. Twenty-two patients with nodular-bronchiectatic nontuberculous mycobacterial pulmonary disease were randomized to receive placebo or SPR720 at doses of 500 mg or 1000 mg once daily for 56 days. No overall difference between treatment groups was observed in the primary sputum bacterial burden or time-to-positivity analyses, and no consistent dose–response relationship was demonstrated [19]. Sustained culture negativity occurred in only two of 16 SPR720-treated patients in the microbiological intention-to-treat population, while increased bacterial burden was observed after treatment cessation. Furthermore, reversible hepatic enzyme elevations occurred in 11 of 17 SPR720-treated patients (65%), including three patients (18%) with grade 3 toxicity [19]. Consequently, development of oral SPR720 for MAC pulmonary disease was discontinued [19]. These findings suggest limited bacteriostatic activity under the conditions studied and highlight important safety and efficacy limitations.

Epetraborole is a benzoxaborole antimicrobial that inhibits bacterial leucyl-tRNA synthetase, thereby interfering with protein synthesis. Although its distinct mechanism of action generated interest in its potential use against MAC pulmonary disease, advancement into clinical development should not be interpreted as evidence of established therapeutic efficacy. The Phase 2/3 EBO-301 clinical development program for treatment-refractory MAC pulmonary disease was discontinued in August 2024 following disappointing efficacy results [20]. In a subsequent announcement on 1 May 2025, the sponsor reported that the truncated phase 3 study, which included 97 participants, did not meet its primary endpoint in the Quality of Life-Bronchiectasis (QOL-B) respiratory domain [21].

Telacebec (Q203), an imidazopyridine amide that inhibits the mycobacterial cytochrome bc1 complex, represents another approach to disrupting bacterial energy metabolism. Although its mechanism differs from conventional MAC antimicrobials, evidence supporting its clinical use in MAC pulmonary disease remains insufficient. Its activity against MAC, optimal combinations, and clinical safety require further investigation before its potential role in treatment can be established [4,17].

Collectively, these agents demonstrate both the opportunities and challenges associated with developing new treatments for MAC pulmonary disease. Clofazimine has been evaluated in a randomized MAC-PD treatment study, whereas SPR720 has demonstrated important efficacy and safety limitations in a phase 2 trial [18,19]. Other investigational compounds remain supported by varying levels of preclinical and clinical evidence. These findings emphasize that promising laboratory activity does not necessarily translate into improved clinical outcomes and that rigorous MAC-specific evaluation remains necessary before new agents can be incorporated into routine treatment.

### 4.2. Repurposing and Rational Combination Therapy

Drug repurposing has become an attractive strategy for MAC because development of entirely new antimicrobials requires considerable time and financial investment. Existing FDA-approved agents possess established pharmacokinetic and safety profiles, allowing more rapid progression into clinical evaluation [4].

Current repurposing efforts also focus on optimizing multidrug combinations with complementary mechanisms of action and activity across the distinct pulmonary microenvironments occupied by MAC [5].

Importantly, future regimen design increasingly emphasizes shortening treatment duration while minimizing cumulative toxicity. Rather than adding additional antibiotics indiscriminately, contemporary drug discovery seeks combinations with complementary mechanisms of action that improve bacterial killing without increasing adverse events [4,22].

### 4.3. Host-Directed Therapies

Growing recognition that host immune dysfunction contributes to MAC persistence has increased interest in host-directed therapies (HDTs). Unlike conventional antimicrobials, which act primarily on bacterial targets, HDTs aim to modify host biological pathways to enhance antimicrobial defenses, improve immune regulation, or reduce infection-associated tissue injury [23]. Importantly, some investigational interventions may exert both direct antimicrobial and host-mediated effects. These mechanisms should be evaluated separately, and evidence of reduced intracellular bacterial survival alone does not establish that an intervention acts through host immune modulation.

Macrophages represent the principal cellular target for HDT development because they serve as both the primary host cell for MAC replication and the central effector of antimicrobial immunity [7]. Therapeutic strategies aimed at enhancing macrophage antimicrobial activity include stimulation of IFN-γ signaling, modulation of GM-CSF pathways, enhancement of phagosome maturation, promotion of autophagy, and restoration of intracellular oxidative killing mechanisms [7,23]. Such approaches may increase bacterial clearance while simultaneously improving the efficacy of conventional antibiotics.

Several immunomodulatory interventions have demonstrated encouraging preclinical or early clinical results. Cytokine-based therapies, including recombinant IFN-γ, have shown potential benefit in selected patients with impaired cellular immunity, whereas modulation of inflammatory pathways may reduce tissue injury associated with chronic infection. Additional HDT strategies currently under investigation include CFTR modulators to improve mucociliary clearance, agents targeting inflammatory signaling pathways, and therapies that enhance epithelial barrier function [23].

Because host-directed therapies primarily target host biological pathways rather than bacterial proteins, they may exert less direct selective pressure for conventional target-based antimicrobial resistance; however, this potential advantage is mechanism-dependent and has not been established for GSH- or NAC-based interventions in MAC pulmonary disease [23]. Consequently, HDTs are increasingly viewed as complementary components of multidrug regimens rather than replacements for conventional antibiotics.

### 4.4. Advanced Drug Delivery Systems

One of the major barriers to successful MAC treatment is achieving sufficient antimicrobial concentrations within infected pulmonary tissues while minimizing systemic toxicity. Advanced drug delivery technologies therefore represent an increasingly important component of NTM drug development [5].

The most clinically advanced example is amikacin liposome inhalation suspension (ALIS), which encapsulates amikacin within liposomes to facilitate targeted pulmonary delivery [5,6]. Inhaled liposomal delivery is designed to increase local pulmonary exposure to amikacin while limiting systemic exposure. However, the CONVERT trial compared ALIS plus guideline-based therapy with guideline-based therapy alone, rather than with intravenous amikacin; therefore, it does not establish comparative reductions in nephrotoxicity or ototoxicity relative to parenteral treatment [15]. Clinical trials have demonstrated improved culture conversion among patients with refractory MAC-PD receiving ALIS in combination with guideline-based therapy, leading to regulatory approval for treatment-refractory disease [5].

Beyond ALIS, nanotechnology-based delivery systems continue to expand rapidly. Liposomes, polymeric nanoparticles, asymmetric liposomes, inhalable dry powder formulations, and controlled-release systems have demonstrated improved intracellular macrophage delivery and enhanced penetration into pulmonary lesions in preclinical models [24]. These approaches may increase therapeutic efficacy while reducing dosing frequency and cumulative toxicity, although further clinical evaluation remains necessary.

### 4.5. Drug Discovery Platforms and Translational Models

An important challenge in NTM drug development has been the lack of preclinical models that accurately reproduce human disease. Traditional in vitro susceptibility testing often fails to predict therapeutic efficacy because MAC survives within macrophages, biofilms, and granulomatous lesions that cannot be fully replicated under laboratory culture conditions [7].

Recently, zebrafish infection models have emerged as powerful translational platforms for NTM drug discovery. Zebrafish embryos permit real-time visualization of granuloma formation, macrophage recruitment, bacterial dissemination, and therapeutic responses while maintaining relatively high experimental throughput. Importantly, these models reproduce many pathological features observed during human NTM infection, including intracellular persistence, granulomatous inflammation, and host immune responses [23].

Beyond evaluating direct antimicrobial activity, zebrafish models facilitate simultaneous assessment of host-directed therapies, toxicity, pharmacodynamics, and combination regimens within an intact vertebrate immune system [23]. Consequently, these systems increasingly serve as intermediate platforms bridging in vitro screening and mammalian efficacy studies, accelerating identification of promising therapeutic candidates for subsequent clinical development.

**Figure 2:**
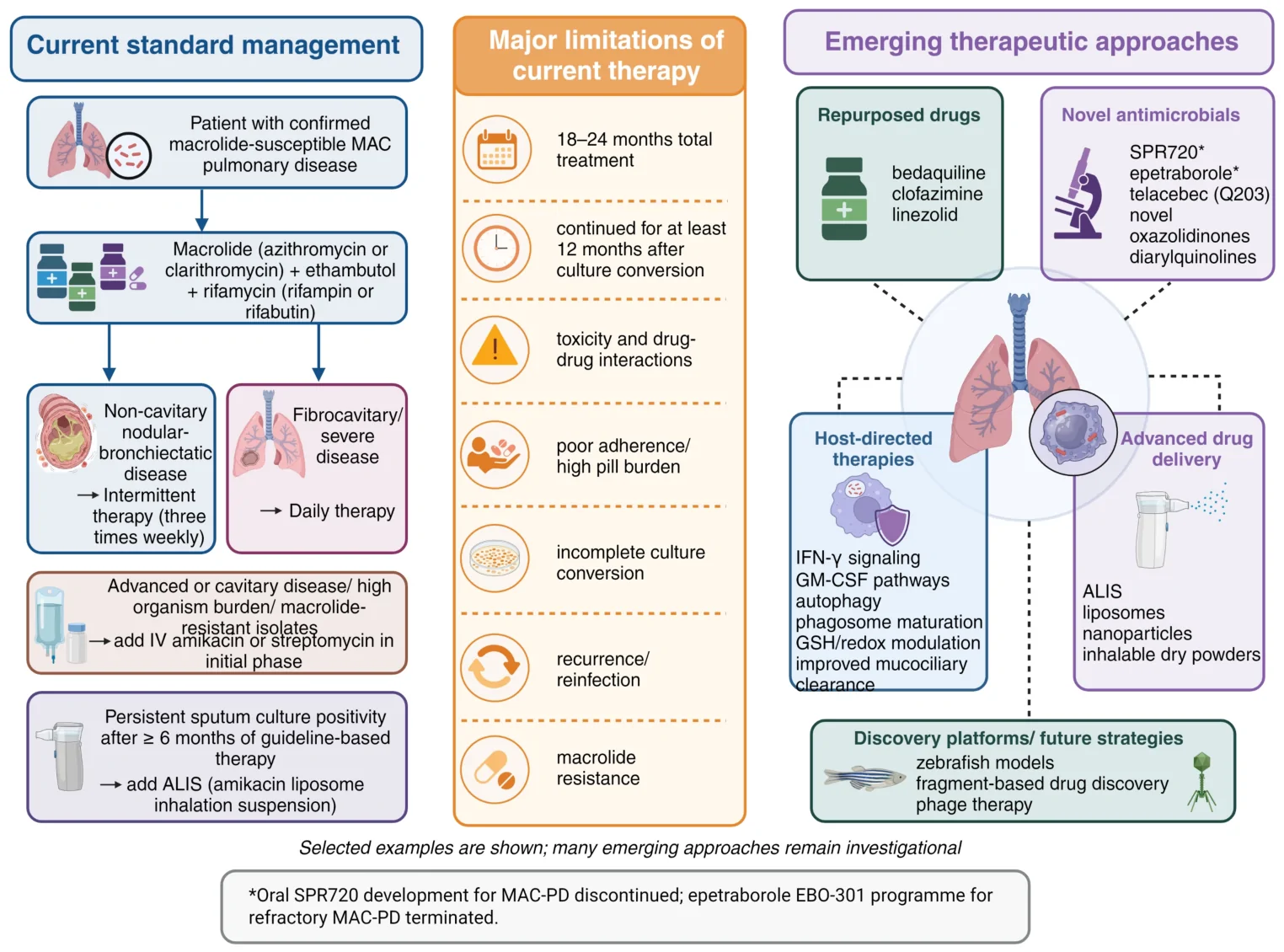
Current and emerging treatment approaches for *Mycobacterium avium* complex pulmonary disease. Standard treatment for macrolide-susceptible MAC-PD consists of a macrolide, ethambutol, and rifamycin. Intermittent therapy is generally used for non-cavitary nodular bronchiectatic disease, whereas daily therapy is preferred for fibrocavitary or more severe disease. IV amikacin or streptomycin may be added during the initial phase in patients with advanced or cavitary disease, high organism burden, or macrolide-resistance. Amikacin liposome inhalation suspension (ALIS) may be added for refractory disease that remains culture-positive despite guideline-based therapy. Major limitations of current treatment include prolonged duration, toxicity, drug–drug interactions, high pill burden, incomplete culture conversion, recurrence or reinfection, and macrolide resistance. Emerging strategies include repurposed drugs, novel antimicrobials, host-directed therapies, advanced pulmonary drug delivery, and discovery strategies such as zebrafish models, fragment-based drug discovery and phage therapy. Selected examples are shown, and many of these approaches remain under investigation. Solid arrows in the left panel indicate treatment decisions, including dosing frequency and when to add therapy. Black dashed lines connect the categories of emerging approaches to the central MAC-PD illustration, and orange dashed lines separate the individual limitations of current therapy. Created in BioRender. Takhmazian, R. (2026) https://BioRender.com/f4fz10y.

Collectively, emerging therapeutic approaches reflect a fundamental shift in MAC drug discovery. Rather than relying solely on prolonged multidrug antibiotic therapy, contemporary research increasingly integrates novel antimicrobials, rational drug repurposing, host-directed immunomodulation, advanced pulmonary drug delivery, and translational discovery platforms to address the complex biology of MAC infection. These complementary strategies offer the potential not only to improve microbiological cure rates but also to shorten treatment duration, reduce toxicity, and limit the emergence of antimicrobial resistance, thereby transforming future management of NTM disease. Current MAC-PD management, major treatment limitations, and emerging therapeutic approaches are shown in Figure 2. Current and emerging therapies for MAC-PD are summarized in Table 1.

## 5. GSH as an Adjunctive Therapy

The persistent intracellular survival of *M. avium* and the chronic inflammatory response associated with MAC pulmonary disease have prompted interest in therapeutic strategies that may complement conventional antimicrobial treatment. Among these approaches, glutathione (GSH), a major intracellular thiol antioxidant, has attracted attention because of its role in maintaining cellular redox homeostasis and regulating immune responses [25,26]. However, the evidence supporting GSH-based interventions arises from distinct experimental and clinical contexts that must be interpreted separately. MAC-specific studies have demonstrated antimycobacterial activity of N-acetylcysteine (NAC) and liposomal GSH in bacterial cultures and infected macrophage models [27,28]. Additional mechanistic evidence derives from studies of tuberculosis and other mycobacterial infections [26,29], while broader investigations of cellular redox regulation provide a biological rationale for potential immunomodulatory effects [25]. Although these findings justify further investigation, they do not establish that GSH supplementation improves antimicrobial treatment outcomes in patients with MAC pulmonary disease.

### 5.1. Biological Functions of GSH

GSH is a tripeptide composed of glutamate, cysteine, and glycine that exists predominantly in its reduced form (GSH) under physiological conditions [25]. Together with its oxidized disulfide form (GSSG), GSH constitutes one of the principal intracellular redox buffering systems responsible for maintaining protein structure, regulating signaling pathways, detoxifying ROS, and preserving cellular homeostasis [25]. The intracellular GSH/GSSG ratio is widely recognized as an indicator of cellular oxidative stress, with reductions in this ratio reflecting impaired antioxidant capacity and increased susceptibility to oxidative injury [25].

Beyond its antioxidant properties, GSH serves as an important regulator of immune cell function. In macrophages, dendritic cells, neutrophils, natural killer (NK) cells, and T lymphocytes, GSH influences cytokine production, antigen presentation, cellular proliferation, and antimicrobial effector mechanisms [26]. Rather than simply suppressing oxidative stress, GSH helps maintain an appropriate redox environment that permits efficient immune activation while preventing excessive oxidative damage to host tissues [25,26]. Consequently, disturbances in GSH metabolism may impair both innate and adaptive immune responses during chronic infectious diseases.

### 5.2. GSH and Mycobacterial Immunity

Intracellular mycobacteria reside within macrophages where bacterial survival depends, in part, on overcoming host oxidative defenses. As discussed in the previous section, *M. avium* possesses multiple antioxidant enzymes that neutralize macrophage-derived ROS and facilitate long-term intracellular persistence [8]. Restoration of host GSH levels may therefore improve macrophage function by preserving redox balance, supporting mitochondrial function, and maintaining effective antimicrobial signaling pathways rather than by directly increasing oxidative stress [25,26].

Evidence from experimental studies of mycobacterial infections, particularly Mycobacterium tuberculosis, suggests that intracellular GSH availability may influence macrophage antimicrobial responses and immune-cell function. Alterations in GSH homeostasis have been associated with changes in inflammatory cytokine production, including IFN-γ and TNF-α, as well as lymphocyte and natural killer cell responses [26]. These observations provide a mechanistic basis for investigating whether restoration of GSH levels could influence host responses during MAC infection. However, findings from tuberculosis models should not be interpreted as direct evidence that GSH supplementation enhances macrophage-mediated clearance of MAC or improves clinical outcomes in MAC pulmonary disease. MAC-specific studies are needed to establish whether these proposed immunomodulatory effects occur in relevant pulmonary infection models.

**Figure 3:**
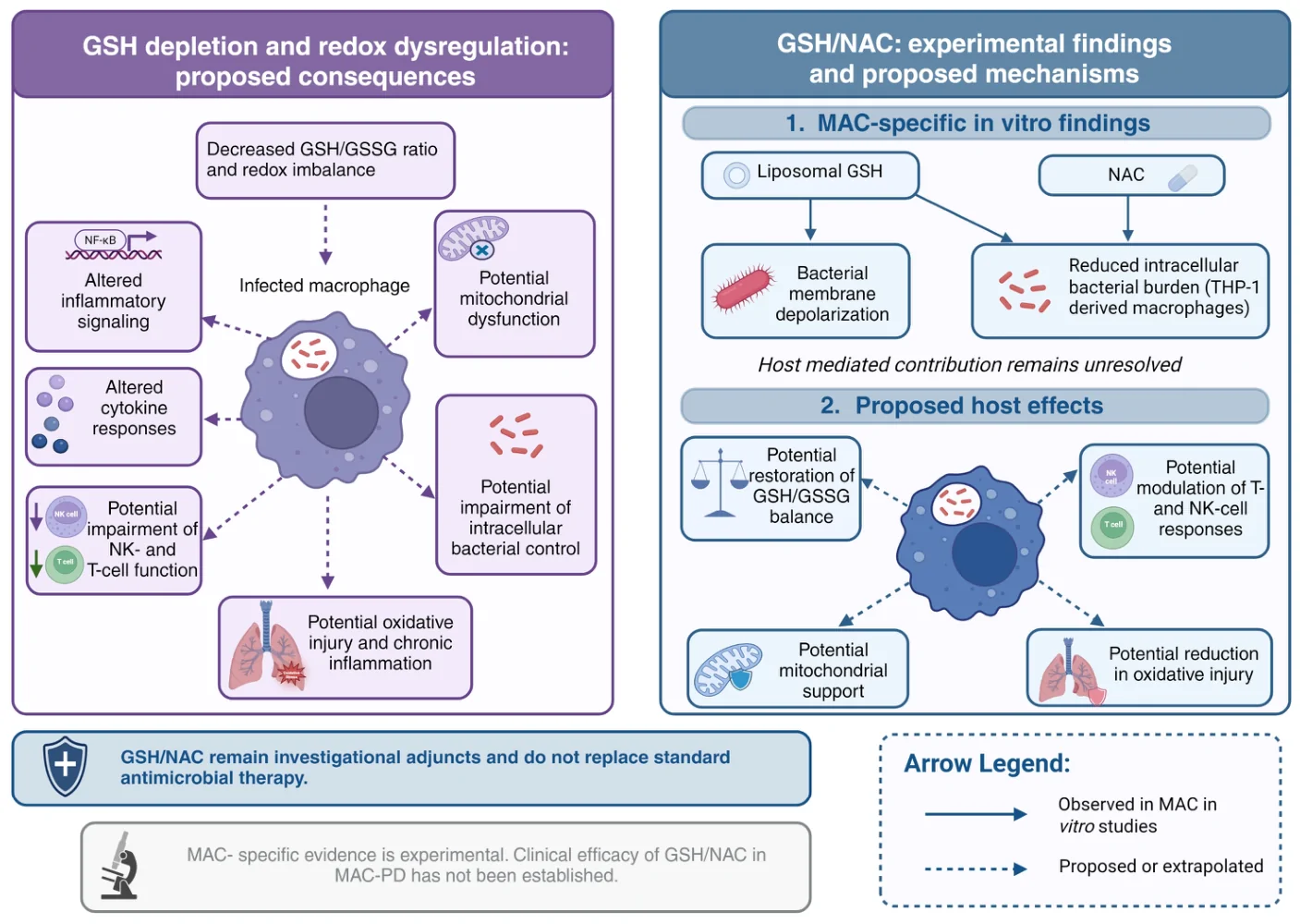
Experimental findings and proposed effects of glutathione-related interventions in *Mycobacterium avium* complex pulmonary disease. The left panel illustrates proposed consequences of GSH depletion and redox imbalance, including altered inflammatory signaling, cytokine responses, immune-cell function, mitochondrial function, and intracellular bacterial control, based on general redox and tuberculosis literature [25,26]. The upper right panel summarizes MAC-specific in vitro findings: bacterial membrane depolarization following liposomal GSH exposure [28] and reduced intracellular bacterial burden in THP-1 derived macrophages following liposomal GSH or NAC treatment [27,28]. Reduced intracellular bacterial burden does not independently establish a host-mediated mechanism. The lower right panel depicts proposed effects on redox balance, mitochondrial function, immune responses, and oxidative injury, extrapolated from general redox and tuberculosis research [25,26]. Solid arrows indicate findings demonstrated in MAC-specific in vitro studies; dashed arrows indicate proposed or extrapolated relationships. GSH/NAC remain investigational adjuncts and do not replace standard antimicrobial therapy. Clinical efficacy in MAC-PD has not been established. Created in BioRender. Takhmazian, R. (2026) https://BioRender.com/jz2oua7. Abbreviations: GSH, reduced glutathione; GSSG, oxidized glutathione; NAC, N-acetylcysteine; MAC, *Mycobacterium avium* complex; MAC-PD, MAC pulmonary disease; NK, natural killer; NF-κB, nuclear factor kappa B.

Beyond its potential effects on antimicrobial immunity, GSH may influence inflammatory signaling through its established role in cellular redox regulation. Persistent mycobacterial infection can generate inflammatory responses and oxidative stress that contribute to pulmonary tissue injury [9]. At the cellular level, GSH participates in maintaining redox homeostasis and regulating signaling pathways associated with oxidative stress and inflammation [25]. These established biological functions provide a rationale for investigating whether GSH restoration could reduce inflammation-associated tissue injury during MAC pulmonary disease. However, the ability of GSH supplementation to preserve pulmonary function, reduce inflammatory damage, or improve bacterial clearance in patients with MAC-PD has not been demonstrated. Accordingly, the relationships between GSH availability, immune regulation, and pulmonary outcomes should be regarded as proposed therapeutic mechanisms rather than established clinical effects. These hypotheses, together with the available experimental evidence, are summarized in Figure 3.

### 5.3. Experimental Evidence and Mechanistic Interpretation

#### 5.3.1. Direct Antimycobacterial Activity

Recent MAC-specific experimental studies have investigated the direct antimycobacterial activity of N-acetylcysteine (NAC) and liposomal glutathione (L-GSH) against *M. avium*. Sasaninia et al. evaluated NAC alone and in combination with the cyclic antimicrobial peptide [R4W4], reporting minimum inhibitory concentrations (MICs) of 5 mM for NAC and 6 µg/mL for [R4W4] under their experimental conditions [27]. NAC and [R4W4] each induced bacterial membrane depolarization, with greater effects observed for selected combinations. Bacterial colony-forming unit assays also demonstrated reductions in *M. avium* survival, although the magnitude and statistical significance of combination effects varied by concentration and assessment time [27]. Newman et al. separately investigated L-GSH and reported an MIC of 0.8 mM, defined in their assay as the lowest concentration producing reproducible growth inhibition of at least 50%; inhibition at this concentration was 67.81% ± 2.45% [28]. L-GSH also reduced bacterial survival in culture and induced membrane depolarization, supporting a potential direct antimycobacterial mechanism [28]. However, these findings were obtained under laboratory conditions, and neither the effective pulmonary exposure nor the clinical safety and efficacy of these concentrations has been established in patients with MAC pulmonary disease. Furthermore, the L-GSH study did not include an empty-liposome control, preventing definitive attribution of the observed membrane effects to glutathione rather than the liposomal formulation [28].

#### 5.3.2. Intracellular Activity and Potential Host-Mediated Effects

Both studies also examined antimycobacterial activity in *M. avium*-infected THP-1-derived macrophage models [27,28]. Sasaninia et al. reported significant reductions in intracellular bacterial counts following NAC treatment at 10 mM (*p* < 0.05) and 20 mM (*p* < 0.0001) after three hours, with additional activity assessed at four and eight days [27]. Selected NAC–[R4W4] combinations further reduced intracellular bacterial survival, although the observed effects depended on the concentrations and time points examined [27]. Newman et al. evaluated L-GSH concentrations ranging from 1 to 40 mM in infected THP-1-derived macrophages and reported reductions in intracellular *M. avium* survival [28]. These findings demonstrate activity in experimental infected-cell systems but do not independently establish whether bacterial reductions result from direct antimicrobial effects, altered macrophage responses, or both. In addition, the L-GSH study did not include a formal dose-dependent macrophage cytotoxicity assessment, particularly limiting interpretation of results at higher concentrations [28]. Neither study established whether the tested concentrations can be achieved safely in human pulmonary tissues or whether these interventions interact beneficially with standard MAC antimicrobial regimens. Further studies using primary human macrophages, clinically relevant bacterial isolates, and appropriate viability and formulation controls are needed before host-directed or clinical therapeutic benefits can be inferred.

#### 5.3.3. Redox Regulation and Inflammatory Modulation

The potential host-directed effects of GSH-related interventions are supported primarily by broader investigations of cellular redox biology and experimental studies involving tuberculosis. GSH participates in cellular redox homeostasis and the regulation of signaling pathways relevant to immune responses and inflammation [25]. Studies of tuberculosis have also explored relationships between GSH availability and antimycobacterial immune function [26,29]. These findings provide a mechanistic rationale for investigating whether GSH restoration could modify macrophage responses or infection-associated inflammation during MAC pulmonary disease. However, the extent to which these mechanisms operate in MAC infection, and whether they translate into improved clinical outcomes, remains uncertain.

Importantly, clinical evidence from tuberculosis does not consistently demonstrate therapeutic benefit from adjunctive NAC despite its effects on glutathione availability. In a randomized clinical trial involving 140 adults with pulmonary tuberculosis, Wallis et al. evaluated NAC at 1200 mg twice daily as an adjunct to standard antituberculosis therapy. Although NAC increased blood glutathione concentrations, it did not significantly improve the time to stable sputum culture conversion (hazard ratio, 0.84; 95% confidence interval, 0.59–1.20; *p* = 0.33) [30]. This negative clinical result underscores the distinction between biochemical or mechanistic effects and demonstrated therapeutic efficacy. Moreover, because tuberculosis and MAC pulmonary disease differ in pathogen biology, host characteristics, and treatment regimens, these findings cannot establish the efficacy or inefficacy of NAC in MAC-PD. Instead, they reinforce the need for adequately designed MAC-specific clinical trials.

An additional limitation concerns the populations in which supporting evidence has been generated. Findings from tuberculosis studies or investigations involving HIV-associated immunodeficiency cannot be assumed to apply directly to patients with MAC pulmonary disease, who frequently present with chronic structural lung abnormalities, including bronchiectasis, and may not have systemic immunodeficiency [3,9]. Differences in pathogen biology, immune status, pulmonary disease manifestations, and antimicrobial treatment requirements may influence both the mechanisms and clinical effects of GSH-related interventions. Consequently, studies involving patients with tuberculosis or disseminated MAC should be considered supportive biological context rather than evidence of therapeutic efficacy in MAC-PD.

Taken together, the available evidence supports three distinct interpretations: direct antimycobacterial effects demonstrated in MAC-specific experimental systems, reductions in intracellular bacterial survival whose underlying mechanisms require further clarification, and proposed host-directed effects derived largely from broader redox and tuberculosis research. None of these findings establishes clinical efficacy of GSH- or NAC-based adjunctive therapy in patients with MAC pulmonary disease.

### 5.4. Future Perspectives for GSH-Based Therapy

Current experimental evidence provides a rationale for investigating GSH-related interventions as potential adjuncts to conventional MAC pulmonary disease treatment. MAC-specific laboratory studies have demonstrated antimycobacterial activity of NAC and L-GSH, including effects on bacterial viability and membrane potential [27,28]. Separately, studies of cellular redox regulation and tuberculosis-related immune responses suggest that GSH availability may influence host immune function and inflammatory signaling [25,26]. However, these direct antimicrobial and proposed host-mediated effects represent distinct mechanisms that require independent evaluation. Their relative contributions to bacterial clearance, pulmonary inflammation, and treatment outcomes in MAC pulmonary disease have not been established.

The clinical development of GSH-related interventions for MAC pulmonary disease requires several important questions to be addressed. First, the optimal formulation, route of administration, and dosing strategy remain uncertain. Free GSH, liposomal GSH, and GSH precursors such as NAC may differ in their bioavailability, cellular uptake, and ability to achieve therapeutically relevant concentrations in pulmonary tissues. Second, the concentrations associated with antimycobacterial activity in experimental models must be evaluated in relation to achievable and tolerable drug exposure in humans [27,28]. Third, potential adverse effects, host–cell toxicity, and interactions with guideline-recommended antimicrobials require systematic investigation. Finally, prospective MAC-specific clinical trials are necessary to determine whether these interventions provide measurable benefits in sputum culture conversion, treatment tolerability, pulmonary function, recurrence, or health-related quality of life. Until such evidence is available, GSH- and NAC-based interventions should be considered investigational adjunctive strategies rather than established components of MAC pulmonary disease treatment.

Collectively, the available evidence identifies GSH-related interventions as candidates for further investigation in MAC pulmonary disease. Importantly, the available MAC-specific studies involve bacterial cultures and THP-1-derived macrophage models rather than patients with MAC pulmonary disease, and therefore cannot establish clinical efficacy, pulmonary drug exposure, or safety in the intended treatment population [27,28]. Experimental studies have reported direct and intracellular antimycobacterial activity of NAC and liposomal GSH against *M. avium* [27,28], while broader investigations of redox regulation and tuberculosis-related immune responses provide a rationale for studying possible host-mediated effects [25,26]. However, the relative contributions of direct antimicrobial activity, immune modulation, and redox regulation remain incompletely defined. Furthermore, whether these experimental findings translate into improved clinical outcomes, reduced antimicrobial toxicity, or shorter treatment duration is unknown. GSH-based approaches should therefore be evaluated as investigational adjuncts to guideline-based antimicrobial therapy, with particular attention to efficacy, safety, pulmonary drug exposure, and interactions with existing treatment regimens. Host-directed and glutathione-related strategies relevant to MAC pulmonary disease are summarized in Table 2.

## 6. Challenges and Future Directions

Despite increasing recognition of MAC and other NTM infections, therapeutic development remains substantially underdeveloped compared with tuberculosis. While tuberculosis drug discovery has benefited from coordinated funding, validated preclinical models, adaptive clinical trial designs, and a growing drug pipeline, NTM drug development remains fragmented and heavily dependent on repurposed agents rather than NTM-specific compounds [17]. This gap is particularly problematic because MAC pulmonary disease requires prolonged multidrug therapy, is associated with incomplete culture conversion, and often recurs even after apparent microbiological success [1,5,6].

### 6.1. Need for Improved Therapeutics

Future therapeutic development should prioritize agents with activity against intracellular, biofilm-associated, slow-growing, and macrolide-resistant MAC populations. Optimizing combinations with complementary mechanisms may improve bacterial clearance across different pulmonary microenvironments while reducing treatment-related toxicity [5,11].

Resistance remains one of the most urgent barriers to improved treatment. Macrolide resistance is especially consequential because macrolides are central to successful MAC therapy, and loss of macrolide activity is associated with markedly poorer outcomes [3,6]. However, susceptibility testing remains imperfect, with strong clinical correlation mainly for macrolides and amikacin, while MIC interpretation for many companion drugs remains inconsistent. Improved molecular resistance markers, standardized phenotypic testing, and better correlation between susceptibility results and clinical outcomes are essential for precision-guided MAC therapy [7].

### 6.2. Clinical Trial Gaps

Clinical trial design for MAC remains difficult because disease progression is slow, patient populations are heterogeneous, and clinically meaningful endpoints are not fully standardized [2,17]. Sputum culture conversion is widely used, but it does not always predict durable cure, relapse, reinfection, symptom improvement, or long-term pulmonary outcomes [5,6]. Future trials should incorporate microbiological endpoints alongside patient-centered outcomes, including respiratory symptoms, fatigue, functional status, quality of life, radiographic progression, pulmonary function, and recurrence after treatment completion [2,17].

Another major gap is the limited representation of key patient subgroups. Patients with cavitary disease, severe bronchiectasis, advanced age, immunosuppression, HIV-associated MAC, macrolide-resistant disease, or multiple drug intolerances are often those with the greatest therapeutic need but are difficult to enroll in conventional trials [3,6,9]. Dedicated studies in these populations are needed to evaluate alternative antimicrobial regimens, inhaled therapies, and host-directed approaches [23]. For GSH- and NAC-based interventions specifically, clinical investigation should first establish whether the activity observed in experimental *M. avium* models [27,28] can be reproduced at clinically achievable exposures and translated into meaningful therapeutic benefit without unacceptable toxicity or adverse drug interactions.

### 6.3. Future Research

Future research should prioritize the clinical validation of emerging antimicrobial, host-directed, and drug delivery strategies discussed in this review. For GSH-based interventions specifically, MAC-focused studies are needed to establish efficacy, safety, optimal delivery methods, and potential interactions with standard antimicrobial regimens before clinical use can be considered [4,22,31,32].

Overall, the future of MAC therapy will likely depend on moving beyond prolonged empiric multidrug regimens toward mechanism-based, individualized treatment. This will require better biomarkers, validated resistance testing, improved trial endpoints, optimized drug combinations, and translational platforms that reflect intracellular persistence and biofilm-associated disease [7,11,17,22]. Addressing these gaps could shift MAC treatment from chronic disease suppression toward shorter, safer, and more durable therapeutic strategies.

## 7. Conclusions

NTM pulmonary disease, particularly disease caused by *M. avium* complex, has become an increasingly important clinical and therapeutic challenge because of its rising prevalence, prolonged treatment requirements, and suboptimal outcomes with existing regimens. Current standard therapy for macrolide-susceptible MAC-PD relies on multidrug treatment with a macrolide, ethambutol, and a rifamycin, with aminoglycosides or ALIS reserved for severe, cavitary, macrolide-resistant, or refractory disease. Although these regimens remain the foundation of care, they are limited by long treatment duration, frequent adverse effects, recurrence, incomplete culture conversion, and the emergence of macrolide resistance.

Emerging antimicrobial and host-directed strategies offer opportunities to improve MAC-PD management, although their clinical utility varies according to the available evidence. Future progress will depend on rigorous MAC-specific evaluation of these approaches, including their efficacy, safety, and potential integration with existing treatment regimens.

Within this therapeutic framework, glutathione (GSH) and its precursor N-acetylcysteine (NAC) warrant further investigation as potential adjunctive interventions. MAC-specific laboratory studies have demonstrated antimycobacterial activity, while research involving tuberculosis and other experimental models suggests possible effects on cellular redox balance and immune regulation. However, these findings remain preliminary, and the clinical efficacy of GSH- or NAC-based interventions in MAC pulmonary disease has not been established. Well-designed MAC-specific studies are therefore needed to evaluate their safety, therapeutic effectiveness, and potential integration with guideline-recommended antimicrobial regimens.

Overall, improving outcomes in MAC pulmonary disease will require continued optimization of guideline-based antimicrobial regimens, development of effective treatments for refractory disease, and rigorous evaluation of adjunctive therapeutic strategies. GSH- and NAC-based interventions represent experimental candidates within this broader framework. Although MAC-specific in vitro studies have demonstrated antimycobacterial activity, these findings do not establish clinical efficacy or safety in patients with MAC pulmonary disease [27,28]. Furthermore, proposed host-directed benefits, including enhanced immune responses and reduced oxidative or inflammatory injury, are supported primarily by mechanistic and experimental evidence and have not been validated as clinical treatment effects in MAC-PD [25,26,27,28,29]. Future investigations should clarify the relative contributions of direct antimicrobial and host-mediated mechanisms, determine whether effective concentrations can be achieved safely in pulmonary tissues, and evaluate interactions with existing antimicrobial regimens. Ultimately, appropriately designed MAC-specific clinical trials are needed to determine whether GSH- or NAC-based interventions improve microbiological outcomes, treatment tolerability, pulmonary function, or patient-centered measures. Until such evidence becomes available, these approaches should be regarded as investigational adjuncts rather than established components of MAC-PD treatment.

## Figures and Tables

**Table 1:** Current and emerging antimicrobial therapies for *Mycobacterium avium* complex pulmonary disease.

Therapy	Mechanism/Role	Evidence Type and Population	Main Finding	Clinical/ Development Status	Principal Limitation	Ref.
Azithromycin or clarithromycin	Protein-synthesis inhibition; core macrolide	Guideline recommendation/evidence synthesis; MAC-PD	Macrolide susceptibility predicts response	Standard multidrug therapy	Resistance, GI intolerance, QT prolongation, hearing effects	[14]
Ethambutol	Cell-wall inhibition; companion drug	Guideline recommendation/evidence synthesis; MAC-PD	Helps protect macrolide activity	Standard multidrug therapy	Optic neuropathy; visual monitoring	[14]
Rifampin or rifabutin	RNA-polymerase inhibition; rifamycin companion	Guideline recommendation/evidence synthesis; MAC-PD	Standard regimen component	Established therapy, not repurposed agents	Hepatotoxicity and interactions	[14]
Parenteral amikacin or streptomycin	Protein-synthesis inhibition; initial intensification	Guideline recommendation/evidence synthesis; severe MAC-PD	Suggested for cavitary, advanced/severe bronchiectatic, or macrolide-resistant disease	Initial add-on, generally ≥2–3 months	Ototoxicity and nephrotoxicity	[14]
Amikacin liposome inhalation suspension (ALIS)	Inhaled liposomal aminoglycoside; adjunct to background therapy	Phase 3 open-label randomized CONVERT trial; 336 adults with refractory MAC lung disease	Culture conversion ^1^ by month 6: 29.0% (65/224) vs. 8.9% (10/112) with background therapy alone; OR 4.22, 95% CI 2.08–8.57; *p* < 0.001	Established add-on after ≥6 months of unsuccessful guideline-based therapy	Respiratory adverse events: 87.4% versus 50.0%. No direct comparison with IV amikacin	[14,15]
Clofazimine	Membrane/respiratory effects; alternative companion	Single-center, non-blinded randomized trial; 40 adults receiving macrolide-ethambutol	Culture conversion ^2^: 62% (13/21) with clofazimine vs. 58% (11/19) with rifampicin. Diarrhea: 76% vs. 37%, *p* = 0.012; arthralgia 5% vs. 37%, *p* = 0.011	Alternative to rifampicin in selected regimens	Small trial; noninferiority could not be statistically established. Adverse-event profiles differed; results should not be generalized to all MAC-PD populations	[18]
Linezolid; moxifloxacin	Protein-synthesis or DNA-gyrase inhibition, respectively	Guideline-listed alternatives; limited clinical evidence	No established class-wide efficacy estimate	Selected alternatives for intolerance/resistance	Linezolid: cytopenias/neuropathy. Moxifloxacin: variable activity and QT effects	[14]
SPR720	Prodrug of SPR719; DNA gyrase B ATPase inhibition	Phase 2 proof-of-concept study; 25 patients, including 22 randomized; 56-day treatment	No overall separation from placebo on primary microbiological endpoints; post-treatment bacterial rebound. Hepatic-enzyme elevations: 11/17 (65%), including grade 3 toxicity in 3/17 (18%)	Development of oral SPR720 for MAC-PD discontinued because of hepatic safety concerns	Limited bacteriostatic activity, hepatic toxicity, small sample, and short monotherapy exposure	[19]
Epetraborole	Oral investigational antimicrobial; adjunct to optimized background regimen	Randomized EBO-301 phase 2/3 program; treatment-refractory MAC-PD; truncated phase 3 included 97 participants; sponsor-reported results	Phase 2 month-6 culture conversion (key secondary endpoint): 13.2% vs. 10.0%, *p* = 0.64, with epetraborole vs. placebo added to optimized background therapy. Truncated phase 3 did not meet its QOL-B respiratory-domain primary endpoint.	EBO-301 trial for treatment-refractory MAC-PD terminated; announced August 2024.	No demonstrated culture-conversion advantage in phase 2; truncated phase 3 missed its primary endpoint. Sponsor-reported findings have less methodological detail than a full trial publication.	[20,21]

Current and emerging antimicrobial therapies for MAC pulmonary disease: mechanisms, evidence, key findings, development status, and limitations. ^1^ CONVERT: conversion required three consecutive monthly MAC-negative sputum cultures by month 6 [15]. ^2^ Clofazimine trial: conversion required three consecutive negative sputum cultures, with the first occurring within 6 months [18]. These timing rules differ and should not be treated as identical endpoints. For macrolide-susceptible MAC-PD, guideline-based treatment generally continues for at least 12 months after culture conversion; treatment initiation is individualized [14]. Guideline rows summarize recommendations rather than results from a single trial. Abbreviations: MAC-PD, *Mycobacterium avium complex* pulmonary disease; MAC-PD, MAC pulmonary disease; ALIS, amikacin liposome inhalation suspension; OR, odds ratio; CI, confidence interval.

**Table 2:** Glutathione- and N-acetylcysteine-based strategies: experimental MAC evidence and clinical TB context.

Strategy	Mechanism or Proposed Role	Evidence/Model	Key Findings	Main Limitations	Ref.
Liposomal GSH	Direct bacterial effects; proposed host redox modulation	Axenic *M. avium* and THP-1-derived macrophages	Author-reported, assay-defined MIC: 0.8 mM. Axenic CFU decreased at 20–40 mM after 3 h (*p* ≤ 0.0001); below detection at 40 mM by day 8. Membrane depolarization observed.	In vitro evidence; no patients studied. Macrophage cytotoxicity was not formally assessed; formulation contributions remain unresolved. Intracellular activity does not establish immune mediation. ^1^	[28]
N-acetylcysteine (NAC)	Direct antimicrobial activity; GSH precursor	Axenic *M. avium* and THP-1-derived macrophages	MIC: 5 mM. At 3 h, intracellular CFU decreased with 10 mM (*p* < 0.05) and 20 mM (*p* < 0.0001) vs. untreated controls.	No formal post-treatment macrophage cytotoxicity assessment or clinical MAC efficacy data reported. ^1^	[27]
NAC + [R4W4] peptide	Combined direct antimicrobial activity	Axenic *M. avium* and THP-1-derived macrophages	Peptide MIC: 6 µg/mL. Day-4 combinations reduced intracellular CFU vs. peptide alone (*p* < 0.001); 2 µg/mL peptide + 5 mM NAC also outperformed NAC alone (*p* < 0.01)	Effects depend on concentration and comparator; no formal post-treatment cytotoxicity assessment reported. ^1^	[27]
Adjunctive NAC in pulmonary TB	GSH replenishment and redox modulation	Randomized trial; 140 patients; NAC 1200 mg twice daily for 112 days	No significant improvement in stable culture conversion: HR 0.84 (95% CI 0.59–1.20; *p* = 0.33). Blood GSH increased; secondary FVC findings favored NAC. ^2^	TB rather than MAC; open-label trial. Lung-function longitudinal modeling was post hoc; secondary findings require cautious interpretation.	[30]

Glutathione- and N-acetylcysteine-based approaches relevant to MAC pulmonary disease: proposed roles, evidence, key findings, and translational limitations. ^1^ GSH/NAC studies were experimental. Clinical efficacy in MAC-PD has not been established. Achievable pulmonary concentrations, compatibility with macrolides, ethambutol, or amikacin, and resistance-sparing effects remain unestablished. Both studies assessed 3 h, 4 days, and 8 days; NAC-[R4W4] combination experiments used NAC 5–20 mM and peptide 2–8 µg/mL. ^2^ Stable TB culture conversion required two sequential negative specimens collected at least two weeks apart; unsuccessful sputum production despite collection attempts was counted as negative. The overall FEV_1_ estimate had a confidence interval crossing zero. These findings do not establish clinical efficacy in MAC-PD [30]. Abbreviations: MAC, *Mycobacterium avium* complex; MAC-PD, MAC pulmonary disease; NTM, nontuberculous mycobacteria; GSH, glutathione; NAC, N-acetylcysteine; [R4W4], cyclic peptide containing four arginine and four tryptophan residues; MIC, minimum inhibitory concentration; CFU, colony-forming units; HR, hazard ratio; CI, confidence interval; TB, tuberculosis; FVC, forced vital capacity; FEV_1_, forced expiratory volume in one second.

## Data Availability

No new datasets were generated or analyzed in this review. All information discussed was obtained from previously published literature cited in the manuscript.

## Abbreviations

The following abbreviations are used in this manuscript:

ALIS	amikacin liposome inhalation suspension
GSH	reduced glutathione
IFN	γ-interferon gamma
GM-CSF	granulocyte-macrophage colony-stimulating factor
IV	intravenous
MAC	*Mycobacterium avium* complex
NAC	N-acetylcysteine
BMI	body mass index
COPD	chronic obstructive pulmonary disease
HIV	human immunodeficiency virus
IL-12	interleukin 12
RNS	reactive nitrogen species
ROS	reactive oxygen species
TB	tuberculosis
Th1	type 1 helper T cell
TNF-α	tumor necrosis factor alpha

## References

[1] Conyers L.E., Saunders B.M. (2024). Treatment for non-tuberculous mycobacteria: Challenges and prospects. Front. Microbiol..

[2] van Ingen J., Obradovic M., Hassan M., Lesher B., Hart E., Chatterjee A., Daley C.L. (2021). Nontuberculous mycobacterial lung disease caused by Mycobacterium avium complex—Disease burden, unmet needs, and advances in treatment developments. Expert Rev. Respir. Med..

[3] Kumar K., Daley C.L., Griffith D.E., Loebinger M.R. (2022). Management of Mycobacterium avium complex and Mycobacterium abscessus pulmonary disease: Therapeutic advances and emerging treatments. Eur. Respir. Rev..

[4] Cross J., Gargate N., Rahman K.M. (2025). Current Advances in Developing New Antimicrobial Agents Against Non-Tuberculous Mycobacterium. Antibiotics.

[5] Charrier E.S.A., Dassonville-Klimpt A., Andréjak C., Sonnet P. (2025). *M. avium* Complex Pulmonary Infections: Therapeutic Obstacles and Progress in Drug Development. Pharmaceuticals.

[6] Ito M., Koga Y., Hachisu Y., Murata K., Sunaga N., Maeno T., Hisada T. (2022). Treatment strategies with alternative treatment options for patients with Mycobacterium avium complex pulmonary disease. Respir. Investig..

[7] Zimenkov D. (2022). Variability of Mycobacterium avium Complex Isolates Drug Susceptibility Testing by Broth Microdilution. Antibiotics.

[8] Abukhalid N., Islam S., Ndzeidze R., Bermudez L.E. (2021). Mycobacterium avium Subsp. hominissuis Interactions with Macrophage Killing Mechanisms. Pathogens.

[9] Chancharoenthana W., Kamolratanakul S., Rotcheewaphan S., Leelahavanichkul A., Schultz M.J. (2025). Recent advances in immunopathogenesis and clinical practice: Mastering the challenge-managing of non-tuberculous mycobacteria. Front. Immunol..

[10] Park H.E., Lee W., Choi S., Jung M., Shin M.K., Shin S.J. (2022). Modulating macrophage function to reinforce host innate resistance against Mycobacterium avium complex infection. Front. Immunol..

[11] McManus W.R., Mulvey K., Schorey J.S. (2026). Mycobacterium avium biofilms: Mechanism of production, composition, and its role in pathogenesis and drug resistance. Crit. Rev. Microbiol..

[12] Vu A., Glassman I., Campbell G., Yeganyan S., Nguyen J., Shin A., Venketaraman V. (2024). Host Cell Death and Modulation of Immune Response against Mycobacterium tuberculosis Infection. Int. J. Mol. Sci..

[13] Galluzzi L., Vitale I., Aaronson S.A., Abrams J.M., Adam D., Agostinis P., Alnemri E.S., Altucci L., Amelio I., Andrews D.W. (2018). Molecular mechanisms of cell death: Recommendations of the Nomenclature Committee on Cell Death 2018. Cell Death Differ..

[14] Daley C.L., Iaccarino J.M., Lange C., Cambau E., Wallace R.J., Andrejak C., Böttger E.C., Brozek J., Griffith D.E., Guglielmetti L. (2020). Treatment of Nontuberculous Mycobacterial Pulmonary Disease: An Official ATS/ERS/ESCMID/IDSA Clinical Practice Guideline. Clin. Infect. Dis..

[15] Griffith D.E., Eagle G., Thomson R., Aksamit T.R., Hasegawa N., Morimoto K., Addrizzo-Harris D.J., O’Donnell A.E., Marras T.K., Flume P.A. (2018). Amikacin Liposome Inhalation Suspension for Treatment-Refractory Lung Disease Caused by Mycobacterium avium Complex (CONVERT): A Prospective, Open-Label, Randomized Study. Am. J. Respir. Crit. Care Med..

[16] Nguyen V.D., Duong H., Lee M.C., Chen J.-H., Huang W.-C., Chen H.-E., Lin J.-C., Wang J.-Y., Lee C.-H. (2025). Two-drug versus three-drug regimens for treating Mycobacterium avium complex infection: A systematic review and meta-analysis. J. Infect. Public Health.

[17] Dartois V., Dick T. (2024). Therapeutic developments for tuberculosis and nontuberculous mycobacterial lung disease. Nat. Rev. Drug Discov..

[18] Zweijpfenning S.M.H., Aarnoutse R., Boeree M.J., Magis-Escurra C., Stemkens R., Geurts B., van Ingen J., Hoefsloot W. (2024). Safety and Efficacy of Clofazimine as an Alternative for Rifampicin in Mycobacterium avium Complex Pulmonary Disease Treatment: Outcomes of a Randomized Trial. Chest.

[19] Ussery X.T., Bhatt N., Critchley I.A., Wong S.L., Pottage J.C., Melnick D., Hamed K.A. (2026). Randomized Study of the Efficacy, Safety, and Pharmacokinetics of SPR720 for the Treatment of Mycobacterium avium Complex Pulmonary Disease. Sci. Rep..

[20] AN2 Therapeutics, Inc. (2024). AN2 Therapeutics Provides Update on EBO-301 Phase 2/3 Study Evaluating Epetraborole for Treatment-Refractory Mycobacterium avium Complex (MAC) Lung Disease. https://www.businesswire.com/news/home/20240808125351/en/.

[21] AN2 Therapeutics Reports Data from the Phase 3 Portion of Previously Terminated EBO-301 Study and Outlines Future Milestones from Boron Chemistry Pipeline. Nasdaq. https://www.nasdaq.com/press-release/an2-therapeutics-reports-data-phase-3-portion-previously-terminated-ebo-301-study-and.

[22] Johansen M.D., Hamela C., Ding Y., Kremer L. (2026). Zebrafish Models for Drug Discovery and Therapeutic Validation against Non-Tuberculous Mycobacteria. Cold Spring Harb. Perspect. Med..

[23] Anidi I.U., Olivier K.N. (2023). Host-Directed Therapy in Nontuberculous Mycobacterial Pulmonary Disease: Preclinical and Clinical Data Review. Clin. Chest Med..

[24] Al Badri Y.N., Chaw C.S., Elkordy A.A. (2023). Insights into Asymmetric Liposomes as a Potential Intervention for Drug Delivery Including Pulmonary Nanotherapeutics. Pharmaceutics.

[25] Chai Y.C., Mieyal J.J. (2023). Glutathione and Glutaredoxin-Key Players in Cellular Redox Homeostasis and Signaling. Antioxidants.

[26] Abnousian A., Vasquez J., Sasaninia K., Kelley M., Venketaraman V. (2023). Glutathione Modulates Efficacious Changes in the Immune Response against Tuberculosis. Biomedicines.

[27] Sasaninia K., Era I.H., Newman N., Melendez J., Akif W., Sharma E., Nikjeh O., Glassman I., Jiménez C., Sharma N. (2025). Additive Effects of N-Acetylcysteine and [R4W4] Combination Treatment on Mycobacterium avium. Int. J. Mol. Sci..

[28] Newman N., Sasaninia K., Akif W., Dillard K., Jaime D., Nguyen K., Navarrette M., Melendez J., Era I.H., Xu A. (2026). Liposomal Glutathione Exhibits Direct and Intracellular Antimycobacterial Activity Against Mycobacterium avium via Membrane Depolarization in a THP-1 Macrophage Model. Bioengineering.

[29] Nasiri M.J., Lutfy K., Venketaraman V. (2024). Challenges of Multidrug-Resistant Tuberculosis Meningitis: Current Treatments and the Role of Glutathione as an Adjunct Therapy. Vaccines.

[30] Wallis R.S., Sabi I., Lalashowi J., Bakuli A., Mapamba D., Olomi W., Siyame E., Ngaraguza B., Chimbe O., Charalambous S. (2024). Adjunctive N-Acetylcysteine and Lung Function in Pulmonary Tuberculosis. NEJM Evid..

[31] Togre N.S., Vargas A.M., Bhargavi G., Mallakuntla M.K., Tiwari S. (2022). Fragment-Based Drug Discovery against Mycobacteria: The Success and Challenges. Int. J. Mol. Sci..

[32] Bonacorsi A., Ferretti C., Di Luca M., Rindi L. (2024). Mycobacteriophages and Their Applications. Antibiotics.

